# Social Media Meets Big Urban Data: A Case Study of Urban Waterlogging Analysis

**DOI:** 10.1155/2016/3264587

**Published:** 2016-09-27

**Authors:** Ningyu Zhang, Huajun Chen, Jiaoyan Chen, Xi Chen

**Affiliations:** Computer Science and Technology Institute, Zhejiang University, Hangzhou 310058, China

## Abstract

With the design and development of smart cities, opportunities as well as challenges arise at the moment. For this purpose, lots of data need to be obtained. Nevertheless, circumstances vary in different cities due to the variant infrastructures and populations, which leads to the data sparsity. In this paper, we propose a transfer learning method for urban waterlogging disaster analysis, which provides the basis for traffic management agencies to generate proactive traffic operation strategies in order to alleviate congestion. Existing work on urban waterlogging mostly relies on past and current conditions, as well as sensors and cameras, while there may not be a sufficient number of sensors to cover the relevant areas of a city. To this end, it would be helpful if we could transfer waterlogging. We examine whether it is possible to use the copious amounts of information from social media and satellite data to improve urban waterlogging analysis. Moreover, we analyze the correlation between severity, road networks, terrain, and precipitation. Moreover, we use a multiview discriminant transfer learning method to transfer knowledge to small cities. Experimental results involving cities in China and India show that our proposed framework is effective.

## 1. Introduction

With the design and development of smart cities, opportunities and challenges arise at the moment. For this purpose, a huge amount of physical sensor and social media data need to be obtained. Nevertheless, circumstance vary in different cities due to the variant infrastructures and populations, which leads to the data sparsity, Bassoli et al. [1]. For example, because of the huge population and perfect infrastructure, social media data in big cities are relatively easy to obtain. However, small towns have smaller populations and, hence, relatively inactive social media. Therefore, it is difficult to build a smart city system based on those data. Meanwhile, numerous applications have been modeled through the analysis of data in large cities. To this end, we transfer knowledge from big cities to small ones for urban waterlogging disaster analysis.

With the increasing severity of urban waterlogging disasters in some developing countries, such as China and India, urban waterlogging analysis has become a critical component in modern smart city systems, Gupta [2] and Zhang et al. [3]. Accurate analysis of urban waterlogging conditions can significantly help traffic management agencies generate proactive strategies to mitigate congestion, which can help drivers better plan their trips by avoiding routes expected to be congested. Existing research in the area primarily focuses on past and current conditions, as well as sensors and cameras. However, these data are relatively insufficient to plan for the entire city. Thus, there is considerable interest in using social media to detect urban waterlogging without using physical sensors.

With the rapid growth of social media, more and more people are using Twitter, Facebook, and so forth, to communicate their moods, activities, and plans, as well as to exchange news and ideas, Cranshaw et al. [4]. This has created a massive repository containing information inaccessible through conventional media. This repository includes users' messages relating to urban waterlogging conditions in their areas at different times, such as “Deep water on new seven street. Cars unmoved” and “Big road blocks at intersection; tire in deep water”. In large cities, it is viable to have at one's disposal large amounts of data related to urban waterlogging, Yin et al. [5], Quan et al. [6], and Yadav et al. [7]. However, small cities may not produce adequate social media data for this purpose. Moreover, most waterlogging events are caused by poor road networks, low terrains, and high precipitation in a short time, and normally these kinds of data are easily obtained. Moreover, data in different cities have different distributions. For example, different people may post different tweets for the same event because of reginal differences. The same physical condition may also not necessarily lead to the same severity of waterlogging. In such cases, different cities are equivalent to different domains. It would be helpful if we could transfer urban waterlogging knowledge from its local domain to a new one.

Motivated by the uniqueness of the information available on social media and through satellites and the close relationship between this information and the severity of urban waterlogging, we set ourselves the task of determining whether we can retrieve the relevant Twitter and satellite data and transfer the knowledge conveyed by these to small cities to analyze urban waterlogging, Wu et al. [8]. We analyze twitter data to acquire the locations of urban areas affected by waterlogging and determine the severity. We utilize the open APIs to access the stream of observation records and then establish a correlation between the relevant social media content and satellite features. Moreover, we map the location with entities from external knowledge bases to enrich features. Following this, we analyze the waterlogging data and transfer them to small cities, for which we do not have adequate data of this sort, through a multiview discriminant transfer learning method. We found that most small cities can monitor urban waterlogging disasters through our method.

This paper's major contributions can be described in three aspects:

1. We propose a multiview discriminant transfer learning method between cities for urban waterlogging disaster analysis.
2. We analyze the features that have influence on urban waterlogging disaster analysis.
3. We evaluate the method by various data sources including global satellite-based precipitation data, weather forecast reports data, and Weibo/WeChat data in China and India.

The rest of the paper is organized as follows. In Section 2, we briefly review existing work on social media disaster mitigation and data sparsity in urban computing. We offer preliminary definitions and present the problem statement in Section 3. In Section 4, we propose social and physical view analyses as well as the proposed multiview discriminant transfer learning method for urban waterlogging. We show the setup and results of our experiments in Section 5 and conclude the paper in Section 6.

## 2. Related Work

### 2.1. Social Media for Disaster Mitigation

As mentioned in Section 1, researchers nowadays are trying to exploit the wealth of information available on social media for various purposes. For example, there is considerable interest in using social media to detect emerging news or events: in Petrović et al. [9], the authors address the problem of detecting new events from a stream of Twitter posts using an algorithm based on locality-sensitive hashing. In Sankaranarayanan et al. [10], the authors propose a new processing system called “TwitterStand” to capture tweets that correspond to breaking news. The authors in Sakaki et al. [11] investigate the real-time reception of events, such as earthquakes, on Twitter, and propose a probabilistic spatiotemporal model for the target event that can locate the center and the trajectory of the event.

Furthermore, some researchers are investigating the extraction from tweets of information that might be useful in other domains. In Bollen et al. [12], the authors attempted to determine whether public mood correlates with, or is even predictive of, economic indicators. To this end, they first derived collective mood states from large-scale Twitter feeds and then performed a correlation analysis with the Dow Jones Industrial Average (DJIA) over a certain period of time. They showed that the accuracy of DJIA predictions can be significantly improved by including specific public mood dimensions, such as “calm.” In Eisenstein et al. [13], based on geotagged social media, the authors proposed a multilevel generative model that reasons jointly about latent topics and geographical regions.

With the revival of interest in deep learning, incorporating the continual representation of a word as a feature has proved to be effective in a variety of natural language processing (NLP) tasks, such as parsing, language modeling, and named entity recognition (NER). In sentiment analysis, Bespalov et al. [14] initiated word-embedding using Latent Semantic Analysis and represented each of several documents as the linear weight of *n*-gram vectors for sentiment classification. Our proposed work belongs to this direction of research, and we attempt to build a correlation between Twitter data and a new domain, namely, urban waterlogging analysis.

### 2.2. Data Sparsity in Urban Computing

The problem of data-missing was caused by many reasons. For example, different venues have different user visits. More seriously, some venues may not have people visiting them at all. Data sparsity has been studied for many years in research. In urban computing, there have been many techniques that can be applied to tackle this problem. Matrix factorization decomposes a matrix into a production of two or three matrices. When the matrix is very sparse, we usually can approximate it with three low-rank matrices. For more dimensions, tensor decomposition can be used to approximate the tensor with the multiplication of three low-rank matrices and a core tensor. However, these methods can only handle data sparsity in a single city.

There has been a major assumption that the training and testing data must be in the same feature space in machine learning tasks. Nevertheless, this assumption may not hold in many real-world applications, Pan and Yang [15]. For instance, in a classification task, we have insufficient data in one domain of interest but we only have sufficient training data in another domain of interest, which follow a different distribution. Luckily, transfer learning algorithms help solve this problem, which also can deal with data sparsity problems in urban computing.

Transfer learning models data from related but not identically distributed sources. Multiview learning has been studied extensively in single-domain settings, such as cotraining, Dai et al. [16]. However, little has been done with regard to multiview transfer. Chen et al. [17] proposed Cotraining for Domain Adaptation (CODA), a pseudo multiview algorithm with only one view for original data that may not be effective for the real multiview case. Zhang et al. [18] proposed an instance-level multiview transfer algorithm (MVTL-LM) that integrates classification loss and views consistency terms in a large-margin framework. Yang and Gao [19] proposed a multiview discriminant transfer (MDT) learning approach for domain adaptation. Unlike MVTL-LM, our method is at the feature level, which mines the correlations between views together with the domain distance measure to improve transfer. Unlike MDT, our method additionally labels data by social media and is optimized in a mapping algorithm in the urban waterlogging analysis case.

## 3. Approach

### 3.1. Preliminary

**Definition 1 (city block).**

City block is a region divided by a city (e.g., 1 km × 1 km in our experiments), assuming that urban waterlogging severity in different blocks *g* is uniform.

**Definition 2 (social view).**

A feature vector svi of a block which is obtained by various social media assistance data analyses of smart cities:

(1) $$\begin{array}{c} svi=f\left(\;tweets\;\right), \end{array}$$

where *f*(·) is function that converts the block's raw social media data into a feature vector of social view and tweets are the twitter and Weibo (a twitter like website in China) texts with geotags posted in this block.

**Definition 3 (physical view).**

A feature vector pvi of a block obtained from physical sensors:

(2) $$\begin{array}{c} pvi=g\left(\;precipitation,terrain,POIs,road\;\right), \end{array}$$

where *g*(·) is function that converts the block's raw physical sensor data into a feature vector of physical view and precipitation, terrain, POIs, and road are the raw data of precipitation, terrain, POIs, ad road obtained in this block.

### 3.2. Framework

**Figure 1:**
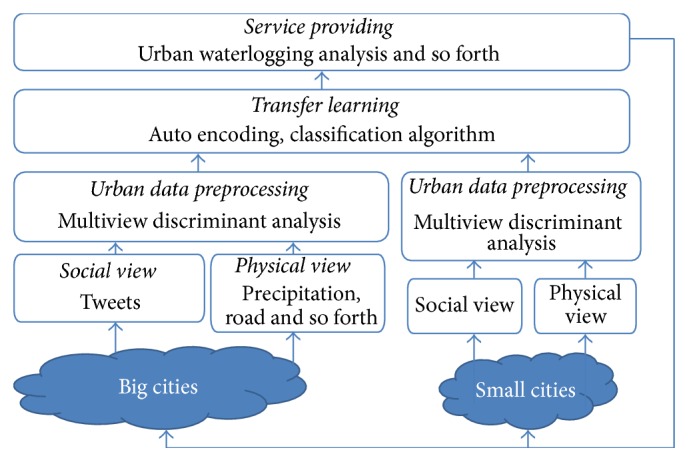
Multiview discriminant transfer learning framework.

As shown in Figure 1, our framework consists of two major parts: feature extraction of the original city and transfer learning, which involves the analysis of urban waterlogging in small cities. We also map the locations of blocks with entities from Yago2 (http://www.mpi-inf.mpg.de/departments/databases-and-information-systems/research/yago-naga/yago/), geoname (http://www.geonames.org/), and WikiData (http://www.wikidata.org) to enrich our waterlogging related knowledge. For example, we may obtain a POI category “Tiandu” for “Residential district.” In this way we may be able to obtain additional knowledge about “Tiandu” and “Residential district”; for example, “what was the place Tiandu before (a lake or lowland may result in severe waterlogging),” or “where is the nearest river to the residential district.” We construct the social view and physical view separately and through multiview discriminant transfer learning we transfer urban waterlogging knowledge to small cities.

*Problem Statement*. Each city contains blocks *C*_*si*_ = {*D*_*s*_}. We use urban sensors and news reported events of each block's waterlogging severity as label. A three-level rating system is adopted: normal (there is no waterlogging at all), middle (the water is very shallow and has no effect on driving), and severe (sever road waterlogging and dangerous for driving). For example, if the sensors or news reporting the location “Changle Road, Hangzhou” have severe urban waterlogging, we calculate the block's id of this location and get the label data “2_15  2”, where the first “2” is the id of “Hangzhou,” “15” is the id of block that contains “Changle Road,”and the last “2” is the label. For the source cities, we have *D*_*s*_ = {(svi^(*i*)^, pvi^(*j*)^, *y* ^(*k*)^)}, where svi^(*i*)^ and pvi^(*j*)^ are social view and physical view of block *i*, *y*_*i*_ ∈ {0,1, 2}.

We utilize FDA2, Diethe et al. [20], to learn a “middle” feature representation *f*_*i*_ of a block *i* from svi^(*i*)^ and pvi^(*i*)^. Then we follow the research of autoencoders, Zhuang et al. [21], to build a feature mapping and use the source domain data jointly for classifier training.

## 4. Transfer Learning Framework

### 4.1. Model Social View

The social media obviously will have huge amount of waterlogging related data for different blocks. So we obtain tweets from twitter and Weibo to obtain features to analysis of urban waterlogging in Indian and China.

We use the trained word-embedding from Glove, Pennington et al. [22]. We manually construct a dictionary D of urban waterlogging with its severity description phrases like {“seeseaontheraod”, “deepwater, carunmoved”,…} and label the phrases. Then we can calculate the average vectors of phases describing normal, middle, and severe waterlogging: Vec_normal_, Vec_midlle_, Vec_severe_. Furthermore, we calculate the top 50 words *W*[50] = {word_1_, word_2_,…, word_50_} that appear in *D* most. For a certain block, now we obtain each word-embedding of tweets posted in this region. We assume that each waterlogging related tweet is true; then we construct the feature vector of social view related to the severity of urban waterlogging. The distance between the words or phases describing the severity can be a good measurement of the real severity of urban waterlogging. For example, some phrases often appear in the place of serious waterlogging such as “see the sea in the downtown,” which means the waterlogging is serious. We represent word-embedding of phases with the average of its word vectors. The word-embedding of phases near these phases describes almost the same severity. Moreover, the frequency of words is also a good feature of event severity. For instance, a block with more than 100 severe waterlogging related tweets may truly have severe waterlogging. Specifically, for a tweet “deep water, car unmoved,” we firstly calculated the vector of tweet through vec_tweet_ = (vec(“deep”) + vec(“water”) + vec(“car”) + vec(“unmoved”))/4; then we calculate the distance of vec_tweet_ and Vec_normal_, Vec_midlle_, Vec_severe_, respectively. At last, we observe weather the words in *W*[50] appear in tweet and record the times.

Finally we build social view of block as
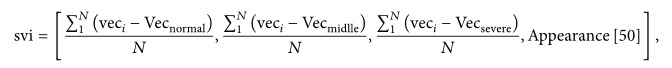
(3)where vec_*i*_ means the word-embedding of a tweet, *N* is the number of tweets in this block, Vec_normal_, Vec_midlle_, Vec_severe_ are average vectors of phases describing normal, middle, and severe waterlogging, and Appearance[50] is a 50-dimensional vector and records the occurrence number of words in *W* (Appearance[1] = 12 means the word *W*[1] appears 12 times in the tweets from this block).

### 4.2. Model Physical View

The concentration of urban waterlogging is influenced by meteorology and terrain. Accordingly, we identify precipitation. We analyze the correlation matrix between urban waterlogging severity and such features using data collected from several cities in China and India. More specifically, for each waterlogging location *l*_*i*_, we measure (1) precipitation, (2) terrain, (3) road network, and (4) POIs by mining the physical features in *g*_*i*_, {*p* : *p* ∈ *P*&*p* ∈ *g*_*i*_} in which *P* is the set of physical view in big cities.

#### 4.2.1. Precipitation

Apparently, the precipitation in specific area and specific period imply the locations and severity of waterlogging. We use the total precipitation data in the last one, two, three, six, twelve, and twenty-four hours and time in the block as features. Formally we have

(4) $$\begin{array}{c} f_{i}^{PR}=\left[\;\left(\;P_{1},P_{2},P_{3},P_{6},P_{12},P_{24},t\;\right)\;\right], \end{array}$$

where *P*_*i*_ is the precipitation in last *i* hours and *t* is the current time.

#### 4.2.2. Terrain

Apparently, a high terrain disperses the concentration of severity, and high precipitation usually causes a high concentration. For example, for a block *g*_*i*_, it has eight neighbours *q*_*i*_. We have to calculate the relative terrain value of block *g*_*i*_ in consideration of *q*_*i*_. For example, a place may have a low terrain value (normally have high possibility of occurrences of waterlogging), but its neighbors are much lower in terrain values, so the possibility of occurrences of urban waterlogging is lower. Formally we have

(5) $$\begin{array}{c} f_{i}^{T}=\left[\;\left(\;\overset{8}{\underset{j=1}{\sum }}\left(\;T\left(\;q_{j}\;\right)-T\left(\;g_{i}\;\right)\;\right),\lambda \;\right)\;\right], \end{array}$$

where *q*_*j*_ is in the block next to *g*_*i*_, *T*(*q*_*j*_) means the elevation of block *q*_*j*_, and *λ* is the elevation measurement error.

#### 4.2.3. Road Network

The structure of a road network has a strong correlation with its terrain pattern, thus providing a satisfactory complement to severity modeling. We identify the following three features for each block based on a road network database: (1) length of elevated road, (2) number of culverts, and (3) the number of intersections in the region. Formally we have

(6) $$\begin{array}{c} f_{i}^{RN}=\left[\;\left(\;L,C,I\;\right)\;\right], \end{array}$$

where *I* is the number of intersections, *L* is the length of elevated road, and *C* is the number of culverts.

#### 4.2.4. POIs

The POIs indicate the patterns of this region, hence contributing to urban waterlogging analysis. A POI may have directly causal relation to it. For example, if a region has large building land spaces, its severity tends to be bad. A park, however, usually leads to less waterlogging. In short, these features are significantly discriminative in urban waterlogging severity analysis. Hence, we apply an entropy to measure the functionality heterogeneity of a block. Let *♯*(*i*, *c*) denote the number of POIs of category *c* ∈ *C* located in *g*_*i*_ and *♯*(*i*) be the total number of POIs of all categories located in *g*_*i*_. The entropy is defined as

(7) $$\begin{array}{c} f_{i}^{POI}=-\underset{c\in C}{\sum }\frac{♯\left(i,c\right)}{♯\left(i\right)}\times \log\frac{♯\left(i,c\right)}{♯\left(i\right)}. \end{array}$$

At last, we have

(8) $$\begin{array}{c} pvi=\left[\;f_{i}^{PR},f_{i}^{T},f_{i}^{RN},f_{i}^{POI}\;\right]. \end{array}$$

### 4.3. Multiview Discriminant Analysis

In reality we now can concatenate social view and physical view into one single view to adapt to the learning setting. However, this concatenation causes overfitting in the case of a small size training sample and is not physically meaningful because each view has a specific statistical property. According to studies of Zheng et al. [23], treating features extracted from different data sources equally do not achieve the best performance. In contrast to single view learning, multiview learning as a new paradigm introduces one function to model a particular view and jointly optimizes all the functions to exploit the redundant views of the same input data and improve the learning performance.

Diethe et al. extended Fisher's Discriminant Analysis (FDA) to FDA2 by incorporating labeled two-view data into the Canonical Correlation Analysis (CCA) framework as follows, Diethe et al. [20] and Melzer et al. [24]:

(9) $$\begin{array}{c} {max}_{\left(w_{s},w_{p}\right)}\frac{w_{s}^{T}M_{w}w_{p}}{\sqrt{w_{s}^{T}w_{s}}\cdot \sqrt{w_{p}^{T}M_{p}w_{p}}}, \end{array}$$

where

(10) Mw=XsTyyTZs,Ms=1n∑i=1nϕsi−μsϕsi−μsT,Mp=1n∑i=1nϕpi−μpϕpi−μpT,

where *w*_*s*_ and *w*_*p*_ are the means of the source data from the two views. The numerator in (9) reflects the interclass distance, which needs to be maximized, while the denominator reflects the intraclass distance, which should be minimized. The above optimization problem is equivalent to selecting vectors that maximize the Rayleigh quotient:

(11) $$\begin{array}{c} r=\frac{\zeta^{T}Q_{w}\zeta }{\zeta^{T}P\zeta }, \end{array}$$

where $Q_{w}=\left(\begin{array}{c} 0\\ M_{w}^{T} \end{array}\begin{array}{c} M_{w}\\ 0 \end{array}\right),P=\left(\begin{array}{c} M_{s}\\ 0 \end{array}\begin{array}{c} 0\\ M_{p} \end{array}\right)$ and $\zeta =\left(\begin{array}{c} w_{s}\\ w_{p} \end{array}\right)$. Note that *Q*_*w*_ encodes the interclass distance, whereas *P* encodes the compound information about the view-based intraclass distances. Further, *ζ* is an eigenvector. Such an optimization is different from FDA2 and facilitates its extension to cross-domain scenarios, which will be presented in the following subsection. For an unlabeled instance, the classification decision function is given by

(12) $$\begin{array}{c} f\left(\;s_{i},p_{i}\;\right)=\left[\;w_{s}^{T}\phi \left(\;s_{i}\;\right)+w_{p}^{T}\phi \left(\;p_{i}\;\right)-b\;\right], \end{array}$$

where *b* is the threshold.

After multiview discriminant analysis, we obtain a single view feature vector for each city, which is able to be used by machine learning algorithm. However, the sparsity of label data is still a problem. The model based on these single city data is quite unreliable. So we try to use transfer learning.

### 4.4. Autoencoders

In our problem, the feature vector of different cities has different distributions. The feature-representation-transfer approach to the inductive transfer learning problem aims at finding good feature representations to minimize domain divergence and classification or regression model error. We use autoencoder to construct a feature representation. An autoencoder is a mapping from an instance *x* to a hidden representation *z* through *z* = *h*(*Wx* + *b*), After that, the hidden representation *z* is reconstructed to $\hat{x}=g(W^{\prime }z+b^{\prime })$.

The object function of autoencoder is formalized as

(13) $$\begin{array}{c} {min}_{W,b,W^{\prime }b^{\prime }}=\overset{n}{\underset{i=1}{\sum }}{\left\|\;x_{i}-{\hat{x}}_{i}\;\right\|}^{2}. \end{array}$$

### 4.5. Transfer Learning

Finally we proposed the optimization problem as follows:

(14) minΘ,Θ′,θj=ϵxS,x^S,xT,x^T+γΩΘ,Θ′+αιzS,yS;θj,

where the first term represents the reconstruction error:

(15) ϵxS,x^S,xT,x^T=∑i=1r ∑i=1njxSi−x^Si2+∑i=1nxTi−x^Ti2,

where *x*_*S*_, *x*_*T*_ are the source and target feature representations and ${\hat{x}}_{S},\;\;{\hat{x}}_{T}$ are the representations through encoding and decoding by autoencoder.

The second term represents regularization: Θ = {*W*, *b*} and Θ′ = {*W*′, *b*′}.

The third term represents the total loss of the softmax regression classifier.

We adopt the gradient descent methods for the solution.

**Algorithm 4 .**

Multiview transfer learning with autoencoders:

*Input*

 The source dataset *D*_*s*_ = {(svi^(*i*)^, pvi^(*j*)^, *y* ^(*k*)^)}
 The target dataset *D*_*t*_ = {(svi^(*m*)^, pvi^(*n*)^, *y* ^(*p*)^)}
 Trade-off parameters *α*, *γ*, hidden features number *k*.

*Output*. Target domain classifier.

(1) Initialize *W*, *b*, *W*′, and *b*′.
(2) Run multiview discriminant analysis to combine social view svi and physical view psi into a single view.
(3) Fix {*θ*_*j*_}; update *W*, *b*, *W*′, and *b*′ alternatively.
(4) Fix *W*, *b*, *W*′, and *b*′; update {*θ*_*j*_}.
(5) If converge, output classifier; otherwise, go to Step (3).

### 4.6. Target Classifier Construction

Given *b*, *W*′, *b*′, *W*, and {*θ*_*j*_}, the classifier *f*_*T*_ can be obtained. Formally, we have

(16) $$\begin{array}{c} f_{T}\left(\;x_{T}\;\right)=\frac{1}{r}\overset{r}{\underset{j=1}{\sum }}\left(\;\theta_{j}^{T}\left(\;g\left(\;Wx_{T}+b\;\right)\;\right)\;\right), \end{array}$$

where *g*(*x*) is the classifier function of softmax regression.

## 5. Experiments

### 5.1. Datasets

Urban waterlogging is one of the most serious hazards in several big cities across the world, especially in China and India. In 2013, hundreds of cities reported being waterlogged for dozens of days at various times. The source code and sample data of experiments can be obtained from https://github.com/zxlzr/UrbanWaterloggingInference. In our experiment, we used the following five real datasets showed in Table 1:

1. Social media: we collected data from both Twitter and Sina Weibo, which is a twitter-like website that was in use across at least 10 cities in China and India in 2013 and 2014.
2. Meteorological data: we collected precipitation meteorological data from the National Oceanic and Atmospheric Administration's (NOAA) web service every hour.
3. POIs: we collected POI data from Baidu Maps for each city.
4. Road networks: the road network data was gathered from Openmaps.
5. Terrain: the terrain data was from Openmaps as well.

**Figure 2:**
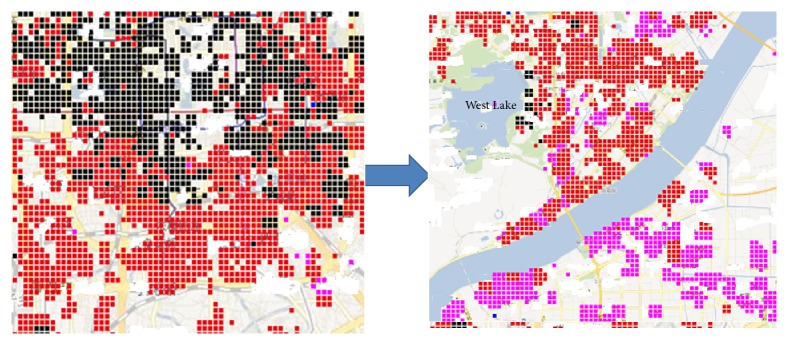
Social media in Beijing and Hangzhou.

The feature data distributions we used in different cities are quite different. As Figure 2 shows, the dark regions mean the waterlogging related social media data are massive. The social media data in Beijing is far more than Hangzhou. Moreover, the label sparsity is quite different. For example, 121 blocks in Beijing (total 200) once have severe urban waterlogging record; however for some relatively small cities such as Hangzhou (total 180) the record is only 65. So we use transfer learning to combine more data. For Hangzhou as a target city and Beijing as source city, we use 30 blocks in Hangzhou and 121 blocks in Beijing of label data as training data, the other 35 as testing data.

### 5.2. Evaluation

In order to get the highest accuracy for all the models, we cross-validate using the development set to find the best hyperparameters. We obtain free-text descriptions of places by adopting geoparsing (https://github.com/ropenscilabs/geoparser) to convert text into unambiguous geographic identifiers (lat-lon coordinates) and map the entities with external knowledge bases. We set the trade-off parameters *α* = 0.01, *γ* = 0.03, and the number of hidden features *k* = 100.

We use different baseline algorithms to verify the effectiveness of our method as follows:

1. *GBRT.* Gradient boosting is a machine learning technique for regression and classification problems. We only use single city data to build a model.
2. *ANN*. We choose Artificial Neural Network (ANN) with backpropagation technique as another baseline. The constructed ANN contains one hidden layer. The ANN method simply treats all labeled data from all stations and all cities (not using multiview) as the training data to build a model.
3. *TrAdaBoost*. Dai et al. [25] proposed a boosting algorithm, TrAdaBoost, which is an extension of the AdaBoost algorithm, to address the inductive transfer learning problems. It attempts to iteratively reweight the source domain data to reduce the effect of the bad source data while encouraging the good source data to contribute more for the target domain and it is an instance-transfer approach.

*Metrics*. Because we use a three-level rating system (2 > 1 > 0), given ReferanceSet obtained from sensors and news report and given PredictionSet = {*y*_*i*_ ≥ 1}, formally we have

(17) Precision=⋂PredictionSet,ReferanceSetPredictionSet,Recall=⋂PredictionSet,ReferanceSetReferanceSet.

We evaluate the final result with *F*1 = (2 × Precision × Recall)/(Precision + Recall) score.

### 5.3. Results

#### 5.3.1. Single City Transfer

We chose a source city and transferred the relevant waterlogging knowledge pertaining to it to a target city. The enhancement obtained by the transfer of learning over the original method is showed in (18). Note that *F*_*st*_ represents the* F*1 score of transfer learning methods from city *s* to city *t*, whereas *F*_*t*_ is the* F*1 score obtained by city *t* itself by GBRT.

**Figure 3:**
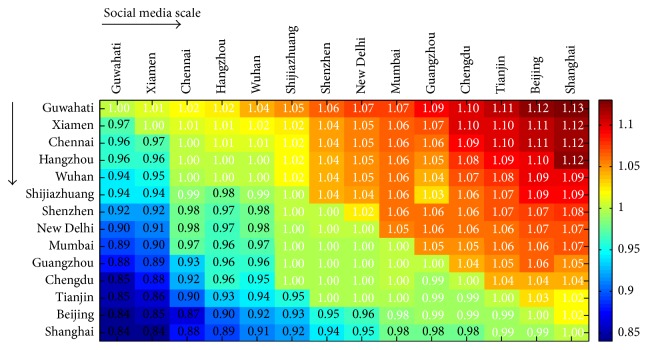
Transfer learning from a single city.

In Figure 3, we show the difference in enhancement between the transfer learning method and the method obtained directly from the city. Different boxes represent the speedup of the transfer learning method and the direct method, from the city represented in the abscissa to that in the ordinate. With the increase in the coordinate axis, the social media size of the city increases, which means the social media size in Beijing is bigger than Tianjin, for example. We use the social media size to evaluate its relative size of the city. In fact, city with more social media activities is usually relatively bigger than that with less social media activities. We see that our transfer learning method outperformed the method obtained directly from the city when transferring knowledge from a large city to a small one. Actually, with the size of social media increasing, the size of training data increases.

(18) $$\begin{array}{c} {Enhancement}_{t}=\frac{F_{\left(st\right)}}{F_{t}}. \end{array}$$

#### 5.3.2. Multiple City Transfer

Finally, we attempted to transfer knowledge from multiple cities to a single city. For example, if Beijing is a target city, we use total cities' data as source city (14 cities). In Table 2, we present the results for all features. Actually, we have observed an improvement compared with the baseline. Regardless of any method, the* F*1 score using total data is better than only using social view or physical view. The GBRT method uses only single city data; its effeteness is the worst. ANN only concentrate all data and do not use multiview method. It is not as good as multiview method. TrAdaBoost and our method use multiview method, while TrAdaBoost is an instance level transfer learning. However, in our problem, the features in different cities have different distribution. So feature-transfer method is better.

## 6. Conclusions

In this paper, we analyzed urban waterlogging using four datasets. We transferred waterlogging knowledge between cities and evaluate our method over 10 cities and a period of more than 18 months. The evaluation showed that transferring urban waterlogging to small cities is applicable.

The transfer learning algorithm proposed here may also have the same effect for some kind of data sparsity, such as AQI in small cities. Thus, we hypothesize that the our algorithm will succeed in transferring other urban knowledge, such as air pollution and traffic from larger to smaller cities and towns with scarce data. This can be understood by analyzing the difference between cities and the rich knowledge transfer obtained from big cities.

In future, we would like to apply our approach to more cities. In addition, we would like to use transfer learning method to solve other data sparsity problems in machine learning.

## Figures and Tables

**Table 1:** Details of the datasets.

Data sources	China	India

City number	10	4
Social media	35,324,237 (Weibo)	1,321,421 (Twitter)
Precipitation	2013.5–2013.12 (7 M)	2013.5–2013.12 (3 M)
Road network	534 (Culvert)	231 (Culvert)
Terrain	437	253
POIs	310,230	75,217

**Table 2:** The results of baseline and transfer learning for five target cities.

Cities	Social view	Physical view	Total

* GBRT *			
Beijing	0.745	0.613	0.815
Shanghai	0.732	0.589	0.712
Hangzhou	0.555	0.511	0.711
Mumbai	0.781	0.631	0.811
New Delhi	0.781	0.711	0.722

* ANN (not multiview)*			
Beijing	0.742	0.675	0.802
Shanghai	0.715	0.688	0.803
Hangzhou	0.713	0.566	0.814
Mumbai	0.832	0.662	0.802
New Delhi	0.754	0.744	0.813

* TrAdaBoost (instance-transfer)*			
Beijing	0.762	0.676	0.812
Shanghai	0.755	0.617	0.883
Hangzhou	0.733	0.555	0.824
Mumbai	0.832	0.722	0.852
New Delhi	0.724	0.784	0.862

* Multiview transfer learning with autocoder (feature-transfer)*			
Beijing	0.753	0.638	**0.852**
Shanghai	0.755	0.687	0.863
Hangzhou	0.763	0.595	**0.894**
Mumbai	0.832	0.732	**0.892**
New Delhi	0.754	0.794	**0.912**
